# Effectiveness of health care workers and peer engagement in promoting access to health services among population at higher risk for HIV in Tanzania (KPHEALTH): study protocol for a quasi experimental trial

**DOI:** 10.1186/s12913-019-4675-z

**Published:** 2019-11-06

**Authors:** Elia John Mmbaga, Germana Henry Leyna, Melkizedeck Thomas Leshabari, Britt Tersbøl, Theis Lange, Neema Makyao, Kåre Moen, Dan Wolf Meyrowitsch

**Affiliations:** 1 Department of Epidemiology and Biostatistics, Muhimbili university of Health and Allied Sciences, P.O.Box 65015, Dar es salaam, Tanzania; 2 Department of Behavioral Sciences, Muhimbili University of Health and Allied Sciences, Dar es Salaam, Tanzania; 3 Department of Public Health, University of Copenhagen, Copenhagen, Denmark; 4 Ministry of Health, Community Development, Gender, Elderly and Children, Dodoma, Tanzania; 5 Department of Community Medicine and Global Health, University of Oslo, Oslo, Norway

**Keywords:** Men who have sex with men, Sex work, Injecting drugs, Health services access, HIV, Tanzania

## Abstract

**Background:**

While there are indications of declining HIV infection rates in the general population globally, Tanzania included, men who have sex with men (MSM), female sex workers (FSW) and people who inject drugs (PWID), now called Key Populations (KP) for HIV epidemic have 2–20 times higher infections rates and contributes up to 30% of new HIV infection. Tanzania have developed a Comprehensive Guideline for HIV prevention among key population (CHIP) to address the epidemic among KPs. However, these populations are stigmatized and discriminated calling for innovative approaches to improve access to CHIP. This project seeks to test the effectiveness of healthcare workers and peer-to-peer engagement in promoting access to CHIP among HIV at risk populations in Tanzania.

**Methods:**

A quasi-experimental design involving Dar es Salaam City as an intervention region and Tanga as a control region will be done. Using respondent driven sampling, 1800 at risk population (900 from Intervention site and 900 from control site) will be recruited at baseline to identify pull and push factors for health services access. Stakeholder’s consultation will be done to improve training contents for CHIP among health care workers and peers. Effectiveness of healthcare workers training and peer engagement will be tested using a quasi-experimental design.

**Discussion:**

The results are expected to co-create service provision and improve access to services among KPs as a human right, reverse HIV infection rates among KPs and the general population, and improve social and economic wellbeing of Tanzanian.

**Trial registration:**

Retrospectively registered on 28th August, 2019 with International Standard Randomized Clinical Trial Number (ISRCTN11126469).

## Background

To date, HIV prevention and research in Africa has mainly focused on HIV transmission in the general population. Remarkably little attention has been paid to other population subgroups; and most notably to some of the groups that are now referred to by UNAIDS [1] as *key populations* at risk for HIV (KP). The sparse results from a limited number of studies on KP in sub-Saharan Africa suggest that HIV rates in men who have sex with men (MSM), people who inject drugs (PWID) and female sex workers (FSW) are 2 to 20 times higher as compared to the HIV rates in the general population [2–15]. Sexual relationship between individuals from KP and the general population are very common and these relationships may play a significant role in HIV transmission between the different groups [3, 5, 10, 14]. Access to health services is key in equipping these populations with the necessary tool for prevention, care and treatment. Although access to health services is a human right issue, results from previous studies indicate that access to services is grossly limited among KP due to stigma and discrimination, and due to poor attitudes towards most key populations in the population and health facilities and the illegal nature of their practices [16–19]. The results of a recent quantitative study among MSM in Tanzania, carried out by authors of this protocol indicated that, while access to services is a human right, only 32.2% of men with anal related disease utilized health facilities. About 73.2% of the respondents reported being stigmatized or discriminated when seeking services in public health facilities [3, 20]. Results of a recent integrated bio-behavioral survey, which involved 620 Tanzanian PWIDS, showed that, 90% of the PWIDS who tested HIV positive were unaware of their HIV status indicating a serious lack of prior access to services [4]. Since KP is estimated to account for about 30% of all new HIV infections in Sub-Saharan Africa, it is obvious that sustainable development goal number 3, UNAIDS goal of eliminating HIV epidemic as a public health threat by 2030 will not be realized if these population sub-groups lack access to prevention, care and treatment [6, 14, 15]. In order to fulfil these goals, UNAIDS have developed a consolidated guideline for HIV prevention, diagnosis, care and treatment among KP [21] and this guideline have been adopted in several countries. In December 2014, Ministry of Health, Community Development, Gender, Elderly and Children in Tanzania released an adapted Comprehensive Guideline for HIV prevention among KP (CHIP) which describes three important areas of interventions for KP (prevention activities, treatment and care activities and psychosocial support activities) [22]. However, there is presently no evidence on the best approaches to optimize access to CHIP among HIV at risk populations. Recent studies indicate that co-creation of health service provision through client engagement is effective in promoting communication with health system, access to services and user satisfaction [23, 24]. This approach is one of the primary goal of the WHO 2020 health program for empowerment of clients to achieve better service utilization and cost effectiveness [25]. Evidence on the extent to which KP and health care worker’s engagement to co-create KP-friendly services for KP is limited in Africa and urgent need to inform CHIP scale up.

### Objective

The overall objective of this project is to evaluate the effectiveness of healthcare workers and KP peer engagement in promoting access to CHIP among HIV at risk populations in Tanzania.

The project seeks to test the following hypotheses:

1. An interplay between individual, interpersonal, contextual, structural, and sociocultural and health system factors will influence health service access among KP
2. Co-creation of health service provision through health care workers training and KP peers engagement is effective in improving access to CHIPs for HIV at risk population

## Methods

### Study setting

The study will be conducted in Dar es Salaam (intervention region) and Tanga Region (Control region). The selected regions are based on the results from previous studies which indicate the presence of substantial number of KPs in those regions with high HIV infection rates and limited access to health services [8, 20].

### Trial design

This is a quasi-experimental design with involvement of two parallel groups of KP living in Dar es Salaam (intervention region) and those living in Tanga region (control region). Co-creation of KP friendly services through training of health care workers and engagement of KP peers form the intervention component to be evaluated.

### Study population and eligibility criteria

The study will involve MSM (a man who regularly or occasionally has sex with another man), FSW (a woman who exchange sex for goods or money) and PWIDs aged 18 and above residing in the selected regions (Table 1).

**Table 1:** Participants Inclusion and Exclusion Criteria

Population	Inclusion criteria	Exclusion criteria
Men who have sex with men	1. Have regular of occasional sexual prelateship with other men (man) 2. Had sex with a man during past 3 months 3. Aged 18 and above	1. Refuse to provide informed consent 2. Mentally ill or severely ill 3. Not a resident of participating region (have an address and lived in the region during the past 6 months)
Female sex workers	1. Exchange sex for goods or money 2. Have exchanged sex for goods or money during past 3 months 3. Aged 18 and above	1. Refuse to provide informed consent 2. Mentally ill or severely ill 3. Not a resident of participating region (have an address and lived in the region during the past 6 months)
People who inject drugs	1. Inject illicit drugs 2. Have injected drugs during past 3 months 3. Aged 18 and above	1. Refuse to provide informed consent 2. Mentally ill or severely ill 3. Not a resident of participating region (have an address and lived in the region during the past 6 months)

### Intervention

#### Peer education

A group of peers will be recruited from the members of the KP communities and trained on safe sexual behaviors, behavioral change communication and role modeling. They will be working within already established KP groups in the regions to offer education on the practice of safer sexual behaviors. They will serve as peer mentors in facilitating access to services and linking member of the KP to friendly clinics and services. This modality will deliver HIV intervention activities and care and treatment referral and support as part of the component of CHIP.

#### KP friendly services

KP friendly health professionals (nurses or clinical officer) will be trained in the selected nearby health facilities to serve as a gateway for KPs in the participating regions. Two such persons will be trained in each of the four selected facilities in Dar es Salaam to serve as a gateway to health services for KPs and will be facilitating service delivery to KPs. They will also be responsible for educating other health personnel in the respective facility and facilitate reduction in stigma and discrimination.

### Outcomes

The primary outcome of this study is change in the proportion of KP who report to have accessed health services measured at baseline, 12 months and 24 months following the intervention. (This correspond to months 36 and 48 of the study) when comparing intervention and control region. Secondary outcomes of the intervention include increased practice of safer sexual behavior (measured by increase in the proportion of participants reporting condom use during last sex and decrease in the proportion of participants reporting having sex with 2 or more sexual partners during past 12 months). Cost-effectiveness of the combined interventions will be assessed by incremental cost effectiveness ratio (ratio of change in the cost of CHIP provision by change in the proportion of population accessing health services. Data from KP interviews and health facilities cost records including those related to health care worker and preventive commodities such as condoms shall be collected.

### Participants recruitment timelines

**Fig. 1:**
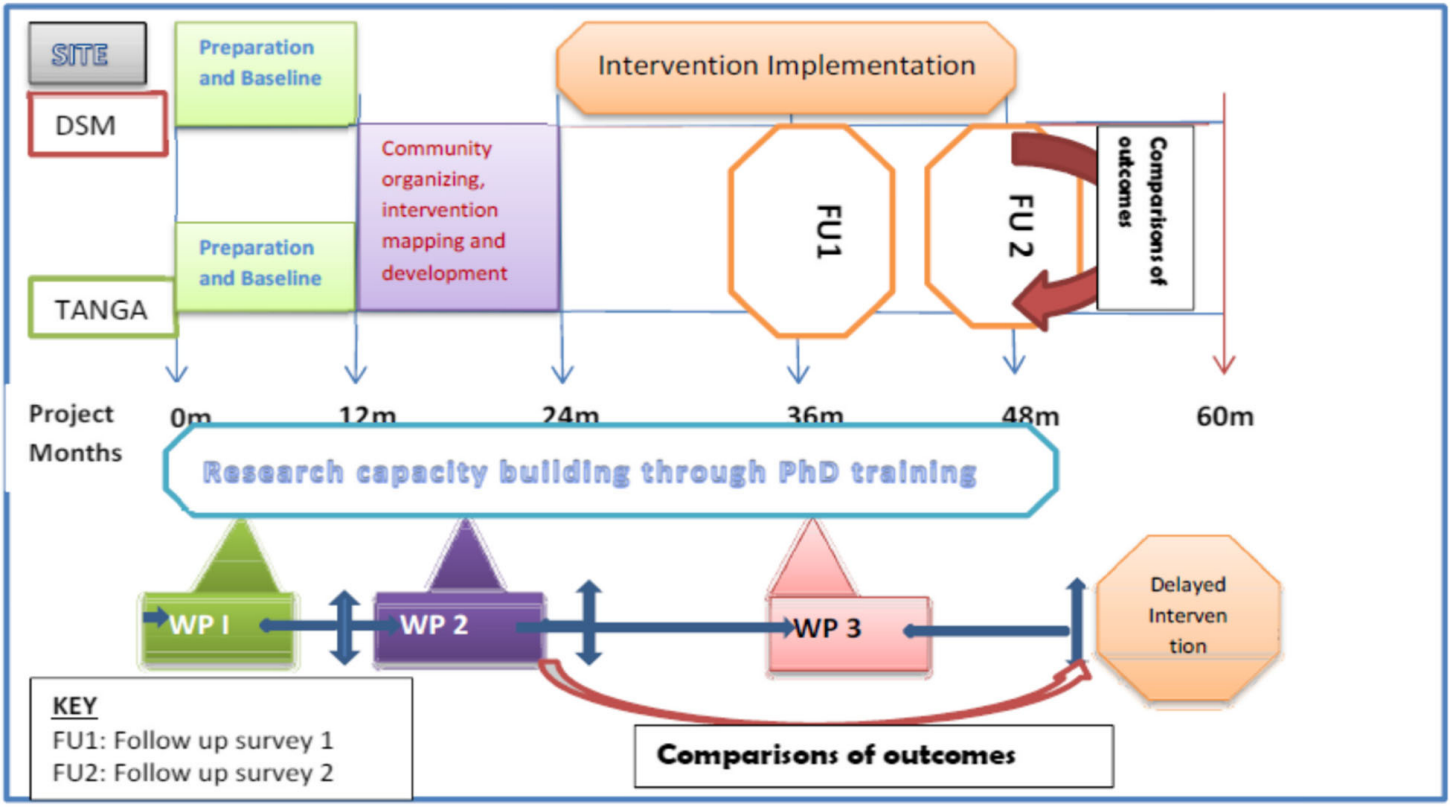
Summary of Project timelines

The study will involve three phases where baseline data collection will take place in year 1 (first 12 months). Intervention mapping activities which will include health facilities, health care workers and KP peer selection, development of KP friendly training materials will be done during year 2. As part of client engagement, all training materials will be developed in collaboration with KP and health care workers and discussed in the community advisory board involving KP. Intervention implementation will take place during month 24 to month 48 with follow up surveys done in month 36 and month 48 (Fig. 1).

### Sample size and power calculations

Assuming 32.2% access to services among KP before the intervention in both sites, increasing to 45% in Tanga and 80% in Dar er Salaam city then 250 participants at each site and each time point will give 80% to detect the treatment effect at the 5% level. To account for drop-out and power loss due to the RDS we increase the sample size to 300 per site and per time point.

### Data collection procedures and methods

A stepwise phased approach will be used in this study where an initial mixed-method study ***(Work package one)*** of the three KPs will be conducted to identify push and pull factors affecting health services access (general health services and CHIP for KP) (Table 2). ***Work package two*** will involve a review of existing comprehensive HIV intervention package using data from WP1 and community advisory board engagement. ***Work package three*** will utilize a quasi-experimental design to assess the effectiveness of the healthcare workers and peer engagement on health service access among KP. All the three phases are interlinked and each previous phase provides data for the next phase (Fig. 1).

**Table 2:** Pull and Push Factors Affecting Health Services Access among Key Populations

Individual	Societal/community	Health system
• Economic status • Socio-demographic characteristics (e.g. education, marital status, age) • Knowledge about CHIP • Individual stigma • Perception of health care worker’s/health system • Attitude towards health care system • Access to health related media • Medical history • Individual experience with health care workers • KP service satisfaction • Experience with violence • Experience with service integration • Opinion on CHIP integration	• Stigma and discrimination • Community perception about KP • Community support to KP • Community opinion on the health needs of KPs • Social connectedness • Community opinions on sharing health services /facilities with members of the KP • Availability of safe KP congregation areas • Community violence and abuse towards KPs	• Distance to health facility • Patient load • Number of health care workers • Health care workers training on sexual and reproductive health issues • Availability of CHIP guidelines • Health care worker’s knowledge on the CHIP guidelines • Health care worker’s perception on KP • Health care worker’s stigma towards KP • Health care worker’s attitude towards KP • Commodity (condom, antibiotics for STI, antiretroviral, contraceptives etc.) stock out • Opinion on KP service integration • Experience with other service integration

### Description of work packages

#### WP 1: (situational baseline assessment)

Both quantitative and qualitative methodologies will be employed in this work package. In the *quantitative component*, a respondent driven sampling (RDS) methodology will be applied to recruit a total of 300 participants from each of the three KPs in Tanga and Dar es Salaam. RDS is a network sampling methodology designed for hard to reach populations where sampling frames are not available. RDS is a method developed for the sampling from populations for which there is no available sampling frame. The method is based on the principle that initial members of the target population termed as “seeds” refer other members of the same population to participate, so that the sample is established by successive generations of recruitment referrals. RDS builds on a mathematical model which provides a theoretical basis for estimation of population proportions and their variances through statistical adjustment [26, 27] Face to face interviews will be used to collect data on individual, societal and health system push factors (drivers) and pull factors (barriers) associated with the use of general and CHIP services. Data on the challenges and opportunity for integration of CHIPs in the existing health services will be collected. The q*ualitative component* will include use Key Informants Interviews (KII) and in-depth interviews (IDI) to collect information on social connectedness and social support; experience of stigma, denouncement, violence and abuse; and access to and utilization of health services. An ecological theoretical model will be used in analysis of the factors at different levels. During this phase, assessment of structural availability for implementation of KPs intervention will be done.

#### WP 2: intervention development

We will work with the KP communities through establishment of community advisory boards and linking with the KP advocacy organization in discussing the results from WP1 and use those results to develop training materials for health care workers and peers. We aim to engage KP to co-create an effective health delivery plan that is responsive to their needs and encourage communication with health care workers. The development strategy will be client promote health services which are client centered and friendly geared towards improved utilization and satisfaction.

#### WP 3: evaluation of effectiveness of intervention

A quasi-experimental design will be used to evaluate the effectiveness of the intervention in improving access to health services as indicated in Fig. 1. The intervention will be implemented in Dar es Salaam region while Tanga region will serve as a control site. Annual assessment using a respondent driven sampling survey will be done at month 36 and 48 after initiation of project in order to assess service utilization indicators [(saturation of KP accessing services measured as a change in proportions of KP reporting use of services over time (saturation proportion over 80%), behavioral change outcome including change in risk behaviors (reduced number of sexual partners, condom use) and cost effectiveness of the intervention.

### Data management and analysis

#### Quantitative data

A special software package for analysis of RDS data (RDSAT) will be used together with STATA version 15. Because selection probability is not the same for each of the participants when RDS is applied, but larger networks are more likely to be represented than small [26, 27], weighting data based on network size will be done by calculating weight as an inverse of the participant’s network size. The primary outcome variable will be change in the proportion of KP reporting to have accessed services during the period of follow up. Weighted point estimates and 95% confidence intervals (CI) will be calculated. Data will be modelled by multiple regressions including site and time point as well as their interaction as covariates. Generalized estimating equation techniques will be employed accounting for correlation due to repeated measure in the analysis of intervention effectiveness. Cost-effectiveness of intervention will be assessed by calculating incremental cost effectiveness ratio (ICER). All the analysis will be two-tailed and significance level will be set at 5%.

#### Qualitative data

Qualitative data analysis will be done inductively following grounded theory procedures. This will allow the study team to follow up and clarify issues that emerge from KII and FGDs as data collection progresses. Data generated will be classified; memo generated according to major themes with assistance from NVIVO 12 software.

### Study feasibility

It is important also to note that MSM, FSW and PWID behaviors are illegal in Tanzania. However, the country regulations allow access to these populations for research and service purposes aiming at improving population health as it has been done by the team of investigators involved in this trial. As a response to the human right and international agreement of “health for all” Methadone Replacement Therapy [28], importation and distribution of condoms for key populations are permitted and advocated by National AIDS Control Programme. It’s with this premise that the new guideline for comprehensive HIV intervention package was developed. We are therefore confident that the experiences of the team and national policies on key population provide a conducive environment for a successful conduct of the proposed project.

### Publication and dissemination strategies

Comprehensive and inclusive dissemination strategies have been drawn for results emanating from this project.

#### Local dissemination

This will involve presentation of project progress and results at the MUHAS research seminars, policy makers as part of the International AIDS Day in December each year and MUHAS Scientific Conference.

#### International dissemination

PhD students to be recruited will write their dissertation in the form of PhD by publications. Each PhD student will publish 4 peer reviewed publications (total 12 publications). Moreover, the results of this project will also be presented in other international conferences such as International AIDS conference, International Conference on AIDS and STI in Africa and IAS Conference on HIV Science.

## Discussion

General guidelines for CHIPs targeting KPs have been issued by WHO [21]. However, this package has not been implemented well in Africa due to lack of evidence on important drivers (pull and push factors) of access to services among KPs. Vertical programs have been condemned by many and efforts to integrate KPs services in the existing health system is among the ten goals agreed by UN high level meeting for HIV prevention [29–32]. This project will therefore engage with KP communities to provide data necessary to inform the improvement of health access for them with them including integration of services in the existing health system. New knowledge to be generated includes: a) Provide information on the determinants of health among KPs; b) Provide data on the most important predictors for health services access; c) Provide evidence on the integration of CHIP in the existing health system; and d) Provide evidence on the effectiveness of Healthcare workers and peer training on access to HIV prevention, care and treatment services among KP.

### Trial status

The trial has received ethical clearance, completed baseline data collection both in the intervention and control regions and intervention implementation is being planned before the end of 2019. Analysis of baseline data to examine the push and pull factors is ongoing and will be completed by 15th September, 2019.

## Data Availability

The datasets used and/or analyzed during the current study are available from the corresponding author on reasonable request.
